# A Single Canal in a Mandibular Second Molar: A Case Report

**DOI:** 10.7759/cureus.24174

**Published:** 2022-04-16

**Authors:** Marwa Alhussain, Jumana Alagil

**Affiliations:** 1 Dental Department, King Abdulaziz Hospital, Ministry of National Guard Health Affairs, Alahsa, SAU; 2 College of Dentistry, Imam Abdulrahman Bin Faisal University, Dammam, SAU

**Keywords:** canal configuration, saudi arabia, second molar, single canal, root canal

## Abstract

Root canal anatomy of mandibular second molars differs among individuals. With the aid of the latest dental technologies in endodontics, the present case report highlights the diagnostic tools required to confirm the morphology of the root canal, and the treatment of uncommon root anatomy of a single-canal single-rooted mandibular second molar. Clinicians should be aware of the various anatomic variations that each tooth may present in order to achieve a satisfactory result. Furthermore, in order to improve the quality of care delivered to their patients, practitioners must have the necessary knowledge and abilities to utilize the diagnostic and therapeutic instruments at their disposal.

## Introduction

The primary goal of endodontic treatment is to prevent and treat microbiological pulpal diseases [1]. Endodontic treatment requires a thorough understanding of the anatomy of root canal systems. Many of the issues that arise before and after root canal therapy are due to the lack of understanding of the anatomy of the pulp complex system [2]. In order to achieve these objectives, the root canal system must be identified, chemo-mechanically cleaned, and shaped [3]. Variations in the form of canal configurations, accessory canals, bifurcation, isthmuses, and anastomoses can be difficult to detect, making endodontic treatment challenging [3]. Inadequate awareness of the root canal system anatomy could be a primary factor in root canal therapy failure [3].

During root canal therapy, some root canals can be missed due to the lack of understanding of the root canal anatomy or the failure to locate additional canals within a complex canal system [2]. Finding all root canals and adequately filling them greatly enhances the therapy outcome [2]. Mandibular molars commonly have two well-defined roots: one mesial root with two canals and one or two canals in the distal root. Endodontic literature has extensively examined the form, configuration, and number of root canals variations in mandibular molars [4]. Moreover, other anatomical variations in mandibular molar teeth have been documented, including the presence of one canal, two canals, four canals, a C-shaped canal, and taurodontism in a single root [4]. Mandibular molar canal configuration has been studied before in the literature. Reuben et al. reported that the prevalence of the presence of a single canal was 1 out of 125 in an Indian population, which brings the prevalence rate to around 1% [5]. Similarly, Weine et al. reported that the prevalence of Vertucci type I canal configuration in second molars was 1.3% [6]. In single-rooted mandibular molars, the mesial canal configuration has been categorized into Vertucci types I, II, IV and V, while the distal root canal configuration is commonly reported to be Vertucci type I [7]. A previously published case report by Alfadley et al. has demonstrated the rare occurrence of a Vertucci type I canal in a second molar in a Saudi Arabian population [8].

A complete understanding of the root canal anatomy and its variations is essential, as it reduces root canal therapy failures. Variations in root canal morphology, especially in teeth with multiple roots are a constant challenge to successful diagnosis and root canal treatment. Morphological changes in canals, such as extra canals, apical deltas, or lateral canals are common, and their frequency and importance are well documented [9]. The purpose of this paper is to report the uncommon canal morphology of a mandibular second molar with a single root and single canal, which has not been commonly reported in endodontic literature.

## Case presentation

A 31-year-old Saudi Arabian woman presented to the dental clinic with a chief complaint of “I want to restore my broken tooth”. The patient pointed to the lower left mandibular area. An assessment of the history of the chief complaint revealed that pulpal therapy for tooth #37 was initiated two years ago and was not completed since. The diagnosis for this tooth is ‘previously initiated pulp therapy with asymptomatic apical periodontitis’. The patient denied any chronic diseases and medical conditions. The patient had reported that they were allergic to eggs. The patient denied any related family history. Informed consent was obtained from the patient for publication of this case.

No significant findings were noted during the extraoral examination. The intraoral examination showed multiple extracted teeth #18, #17, #28, #38 and #36 due to caries without any complications, multiple root canal treatments for teeth #16, #15, #14, #22, #23, #24, #25, #35 and #44, cavitated teeth #16, #15, #14, #13, #12, #11, #21, #26, #27, #34, #43, #44, #46 and #47, and poor oral hygiene. Pulp and periapical tests are summarized in Table 1. The patient had generalized supragingival plaque and calculus. The long-standing edentulous area in the lower-left mandible caused the mesial migration of tooth #37. The periodontal probing depth ranged from 2-4 mm, without tooth noticeable mobility. Moreover, the periodontium was erythematous and bled on probing.

**Table 1:** Intraoral pulpal and periapical examination TTP: tender to percussion/palpation; Not TTP: not tender to percussion/palpation; NR: no response

Test/Tooth	37	35	34
Percussion	TTP	Not TTP	Not TTP
Palpation	Not TTP	Not TTP	Not TTP
Cold	NR	NR	+1/5s

**Figure 1:**
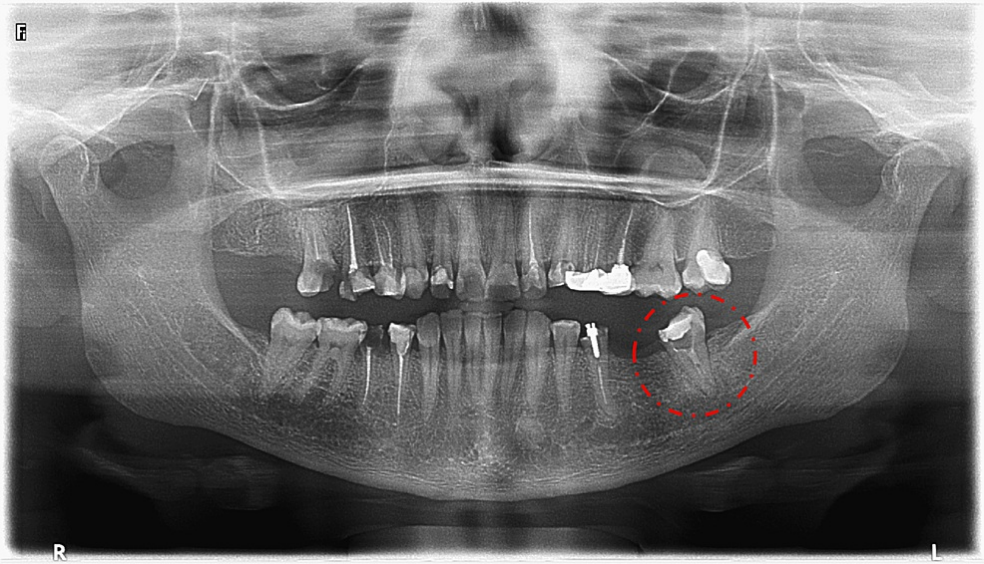
Orthopantomogram (OPG) radiograph

**Figure 2:**
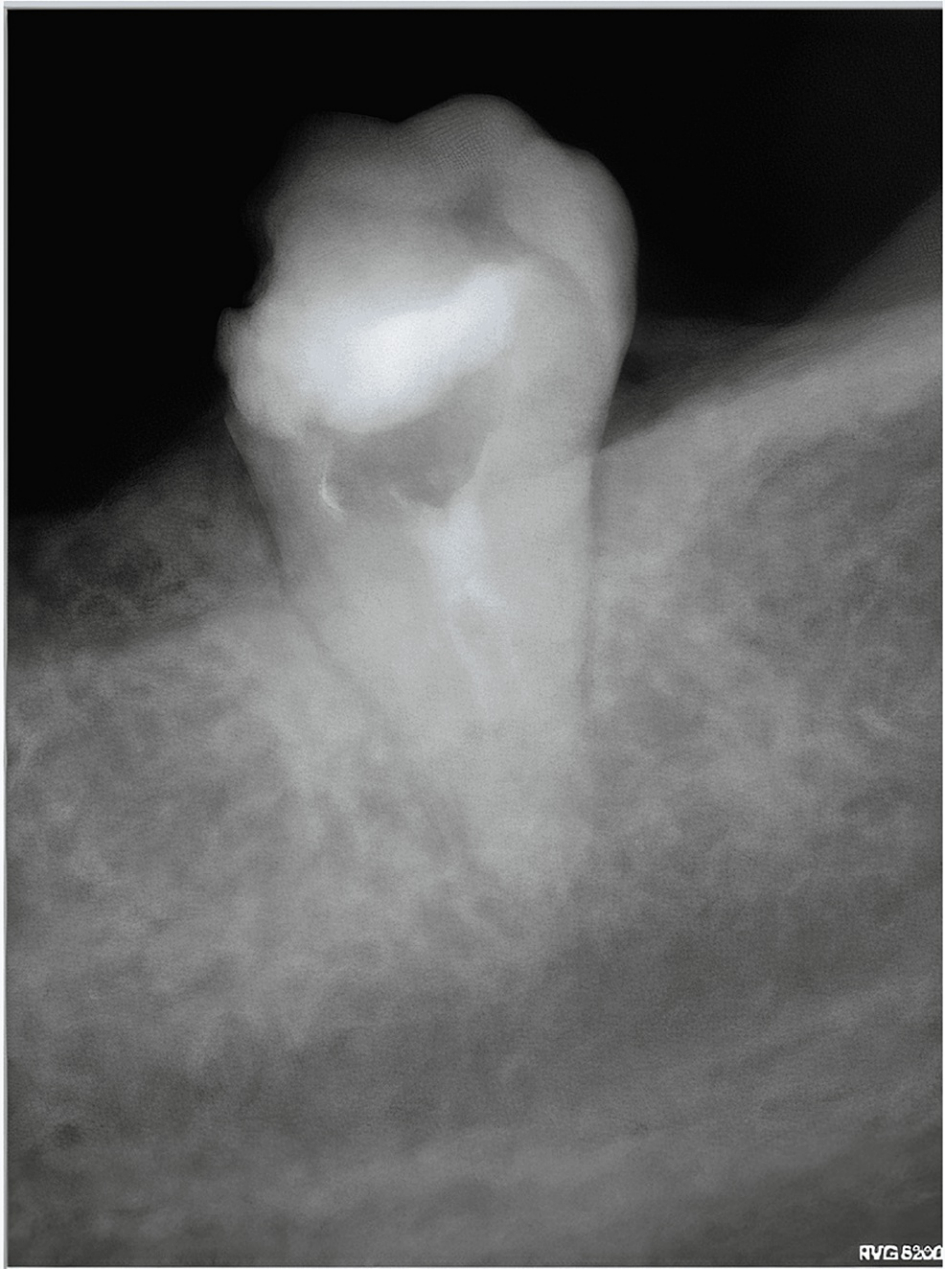
Pre-operative intraoral periapical radiograph demonstrating the radiographical features of tooth number #37 The presence of intracanal medicament is noticed in the radiograph

**Figure 3:**
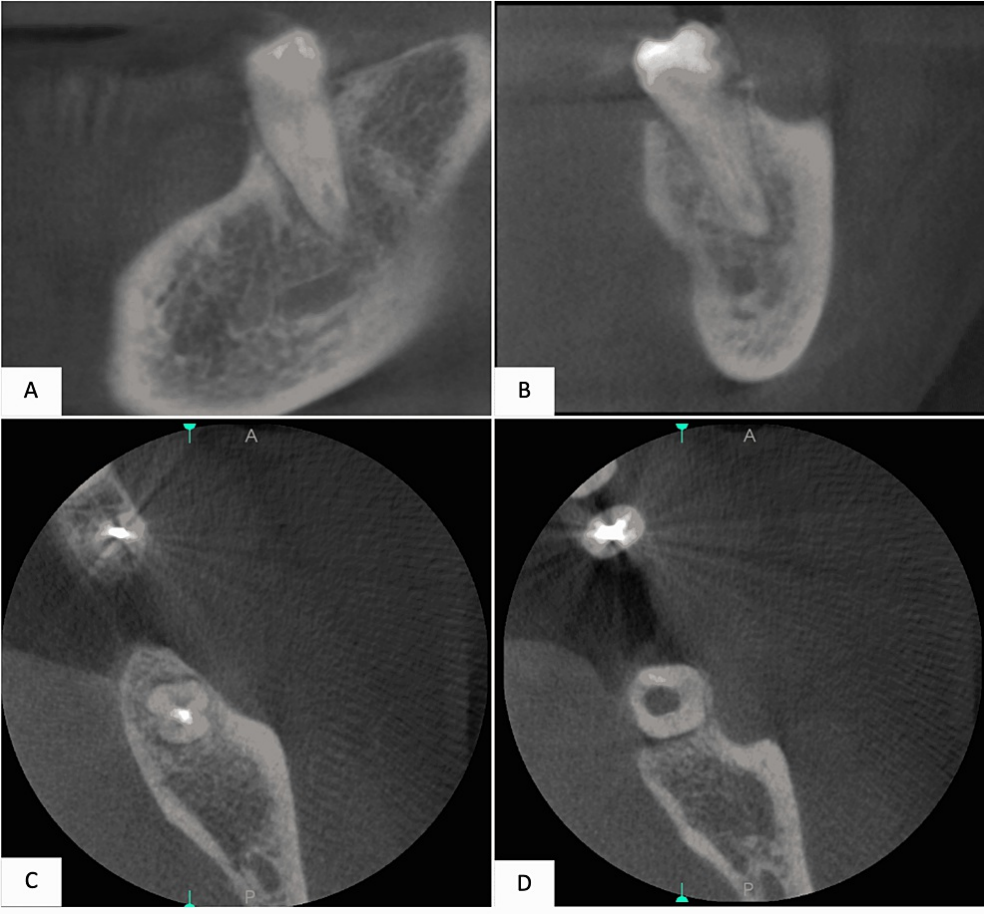
Figure 3: Cone-beam computed tomography (CBCT) for tooth #37 taken at 1 mm slice thickness. Images A and B: Coronal slicing Images C and D: Axial slicing

The radiographic assessment included an orthopantomogram (OPG) image (Figure 1) and a periapical image (Figure 2). Further evaluation of the pulp canal morphology was assessed by Cone-beam computed tomography (CBCT) to ensure a three-dimensional view of the present single canal (Figure 3). The treatment plan of the case was to continue and complete the pulp therapy of tooth #37 as it was a functional tooth with a predicted good prognosis.

**Figure 4:**
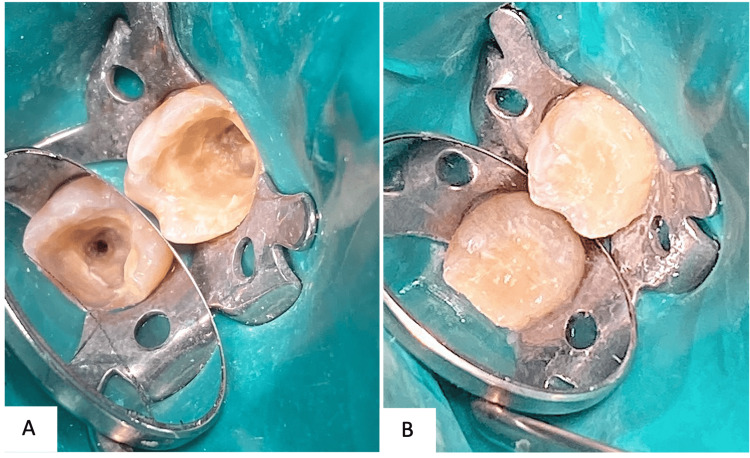
Clinical photographs of tooth #37 A: Straight access cavity established B: Temporary restoration

Treatment was carried out in two visits. During the first visit, the inferior alveolar nerve block was locally anesthetized using one carpule (1.8 ml) of 2% lidocaine with 1:80,000 of epinephrine (LIGNOSPAN SPECIAL 20 mg/ml, Septodont, UK). Another carpule was used to give buccal and lingual infiltration. Tooth isolation was achieved using a rubber dam. A magnification of 3.5X was used. Occlusal glass ionomer temporary restoration was removed. A conventional endodontic access opening was established using endodontic bur size 801 round-diamond-FG with regular-shank in a high-speed handpiece. Access opening was then completed with FG size 1 endodontic bur (Figure 4A). Preliminary radiographs and an iPexII apex locator (NSK-Nakanishi INC, Japan) were used to measure the canal working length. Cleaning and shaping were achieved with the ProTaper Next rotary system (Dentsply Sirona, USA) up to file size F3. The canal was heavily irrigated with 5.25% sodium hypochlorite (NaOCl) and final irrigation was done using ethylenediaminetetraacetic acid (EDTA). Recapitulation and verification of canal patency were performed throughout the pulpal therapy. The tooth was then temporized with a zinc oxide-based restoration (Figure 4B).

**Figure 5:**
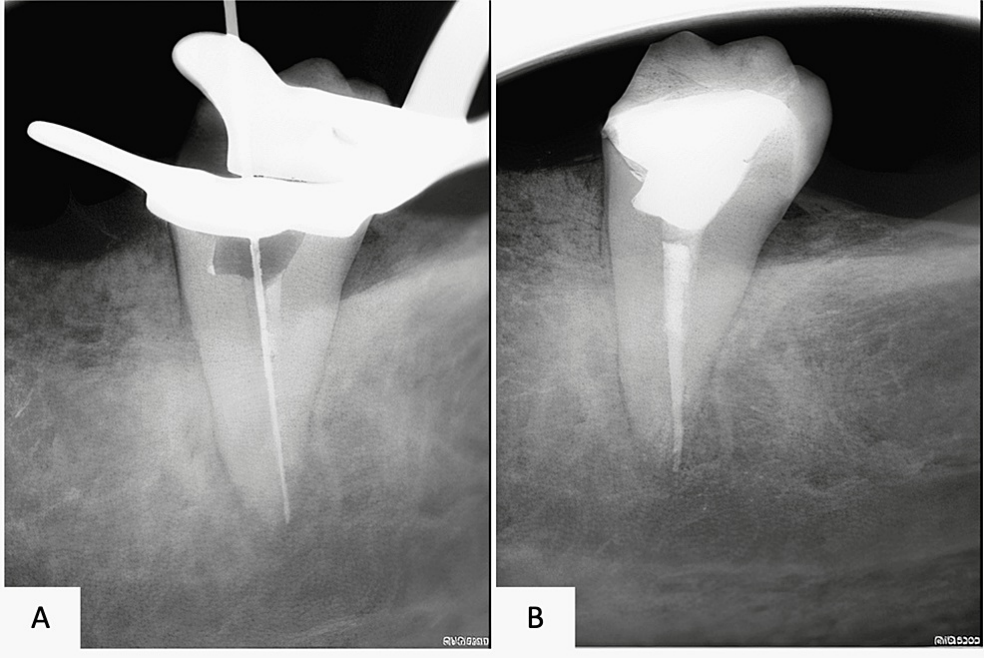
Intraoral periapical radiographs of tooth #37 A: Working length B: Final obturation

During the second visit, The temporary restoration was removed. The canals were dried with sterile absorbent paper points followed by an application of AH plus resin-based sealer. Canals were obturated with a combination of size F3 gutta-percha and warm vertical compaction using the Obtura III Max system (Obtura Spartan Endodontics, USA). Excess gutta-percha was removed using System B (KaVo Kerr, USA). The tooth was temporized with glass-ionomer restoration (Figure 5). The patient was then followed up after one week to report satisfactory completion of the root canal treatment. The patient was then referred to the prosthodontic clinic for the fabrication of a full-coverage restoration.

**Figure 6:**
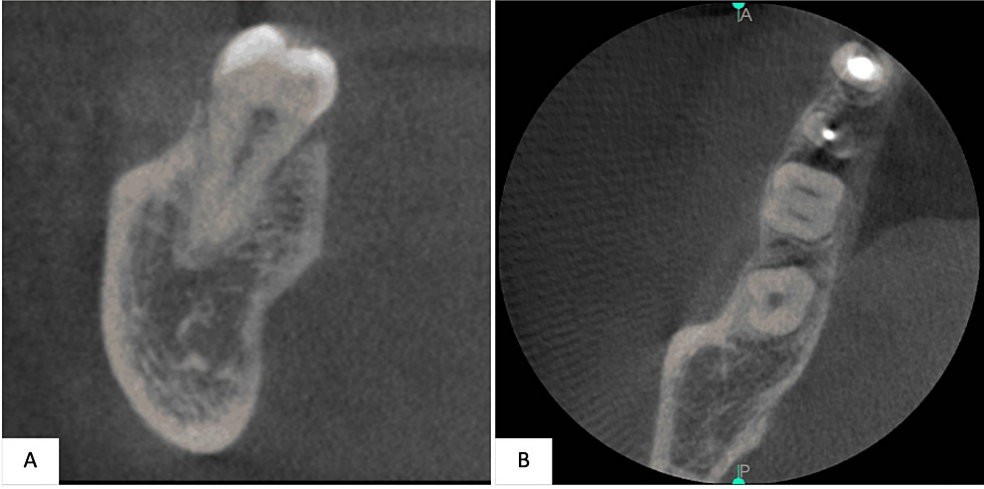
Cone-beam computed tomography (CBCT) for tooth #47 taken at 1mm slice thickness A: Coronal slicing B: Axial slicing

The present case exhibits the bilateral finding incidence, as the contralateral right second molar, tooth #47, radiographically has one single root and one single canal (Figure 6).

## Discussion

The present case report investigates the presence of an uncommon root canal morphology in a mandibular second molar that had a single root and a single canal. A good root canal treatment outcome starts with a well-designed access preparation. Instruments and materials become difficult to use appropriately in a highly complex root canal system without good access. It has been noted that aberrant anatomy can develop in any racial group, depending on a variety of characteristics such as age, sex, and ethnicity, all of which play a part in creating the root canal configuration.

There are multiple tools to help diagnose single canals. These tools include but are not limited to; visual examination under magnification (when available), and different radiography such as cone beam computed tomography (CBCT). Cone beam computed tomography (CBCT) is a safe and non-invasive method for diagnosing and planning root canal therapy. CBCT needs to be requested whenever the evaluation of different angled periapical radiographs fails to offer conclusive information. It also is preferred if buccolingual dimension investigation and analysis are provided. A narrow field of view, which is associated with lower radiation exposure and higher spatial resolution, is advised in circumstances where a CBCT scan is deemed appropriate [10]. This is in an agreement with Kottoor et al. and La et al., as they have recommended the use of CBCT to determine the root canal morphology and canal configurations in cases with aberrations which coincides with the recent update of the American Association of Endodontics (AAE) and American Academy of Oral and Maxillofacial Radiology recommendations [10-13].

Root canal morphology was addressed previously in the literature. For instance, Fava et al. (2000) had published a case report with the same patient exhibiting one root and one canal in all maxillary molars and mandibular second molars [14]. Rahimi et al. (2008) had evaluated the canal configuration of the Iranian subpopulation, it was reported that the incidence of Vertucci Type I was 4.3% [15]. Interestingly, Sabala et al. (1994) reported the bilateral presence of rare findings in an individual [16]. Moreover, Mashyakhy and Gambarini used CBCT to analyze the bilateral symmetry of mandibular first molar roots and root canal systems in a Saudi Arabian population. In the same subject, their symmetrical analysis indicated 100% symmetry in the number of roots, and 56% symmetry in the number of root canals between the right and left teeth [17]. According to the same author, there was no reported gender association with root anatomy and canals morphology based on a study on the Saudi Arabian population [17].

Challenges can arise during root canal therapy of second molars. During the search for missing or additional canals, some of the most common iatrogenic access opening errors occur. Iatrogenic errors can be minimized if the doctor is aware of the pulp chamber's overall location and dimensions. Although extra canals have a higher incidence, the physician should be aware that it is possible for fewer canals to be present than the typically assumed canal shape in some circumstances [18]. Due to the posterior location of second molars, careful access cavity preparation should be taken into consideration to avoid any mishaps. In this case, the mandibular second molar was mesially tilted, which led to distal shifting of the access cavity to avoid mesial perforation.

## Conclusions

The present case report highlights the uncommon anatomy of a mandibular second molar with a single root and single canal. Careful and thorough inspection of the tooth aided with multiple radiographs helps in achieving a satisfactory result for such cases.
